# Deep learning analysis of sinus-rhythm echocardiography for the identification of patients with atrial fibrillation

**DOI:** 10.1093/ehjdh/ztag168

**Published:** 2026-10-21

**Authors:** Ido Cohen, Michael Fiman, Ranel Loutati, Ehud Schwammenthal, Kobi Faierstein, Lior Fisher, Elad Maor

**Affiliations:** Leviev Cardiovascular Institute, Sheba Medical Center, Ramat Gan, Israel; Faculty of Medical and Health Sciences, Tel Aviv University, Tel Aviv, Israel; Aisap.ai, Ramat Gan, Israel; Leviev Cardiovascular Institute, Sheba Medical Center, Ramat Gan, Israel; Faculty of Medical and Health Sciences, Tel Aviv University, Tel Aviv, Israel; Leviev Cardiovascular Institute, Sheba Medical Center, Ramat Gan, Israel; Faculty of Medical and Health Sciences, Tel Aviv University, Tel Aviv, Israel; Aisap.ai, Ramat Gan, Israel; Leviev Cardiovascular Institute, Sheba Medical Center, Ramat Gan, Israel; Faculty of Medical and Health Sciences, Tel Aviv University, Tel Aviv, Israel; Leviev Cardiovascular Institute, Sheba Medical Center, Ramat Gan, Israel; Faculty of Medical and Health Sciences, Tel Aviv University, Tel Aviv, Israel; Leviev Cardiovascular Institute, Sheba Medical Center, Ramat Gan, Israel; Faculty of Medical and Health Sciences, Tel Aviv University, Tel Aviv, Israel; Aisap.ai, Ramat Gan, Israel

**Keywords:** Atrial fibrillation, Echocardiography, Artificial intelligence, Deep learning, Risk stratification, Arrhythmia detection

## Abstract

**Aims:**

Paroxysmal atrial fibrillation (AF) is underdiagnosed due to its intermittent and asymptomatic nature. We evaluated whether a deep neural network (DNN) applied to transthoracic echocardiograms (TTEs) acquired in sinus rhythm can discriminate between patients with and without documented AF and identify individuals at increased risk for subsequent AF documentation and adverse outcomes.

**Methods and results:**

We retrospectively analysed 85 686 TTE studies (2009–21). A DNN was applied to apical four-chamber and parasternal long-axis videos without Doppler. AF ground truth was based on International Classification of Diseases (ICD) codes, electrocardiogram, Holter, or prior echocardiographic documentation of AF. The dataset was split into training (60%), validation (20%), and an independent test set (20%). In the test cohort, we assessed discrimination performance. Among patients without prior AF, we evaluated associations between model classification and subsequent AF documentation and a composite outcome of all-cause mortality, heart failure hospitalization, or stroke. The test set included 17 121 patients (median age 66 years [interquartile range, IQR 54–75]; 57.6% male), of whom 3449 (20.1%) had prior AF. The model achieved an area under the receiver operating characteristic curve of 0.85, with sensitivity 0.80 and specificity 0.72. At the selected threshold, 6581 patients (38.4%) were classified as model-positive, including 3832 (58.2%) without prior AF. Among patients without prior AF, model-positive classification was associated with subsequent AF documentation [adjusted hazard ratio (HR) 1.64; 95% confidence interval (CI) 1.33–2.04] and with the composite outcome (adjusted HR 1.24; 95% CI 1.13–1.35).

**Conclusion:**

Deep learning applied to sinus-rhythm TTE demonstrates discrimination for AF and identifies an imaging-derived signal associated with subsequent AF documentation and adverse outcomes.

## Graphical Abstract

**Graphical Abstract:**
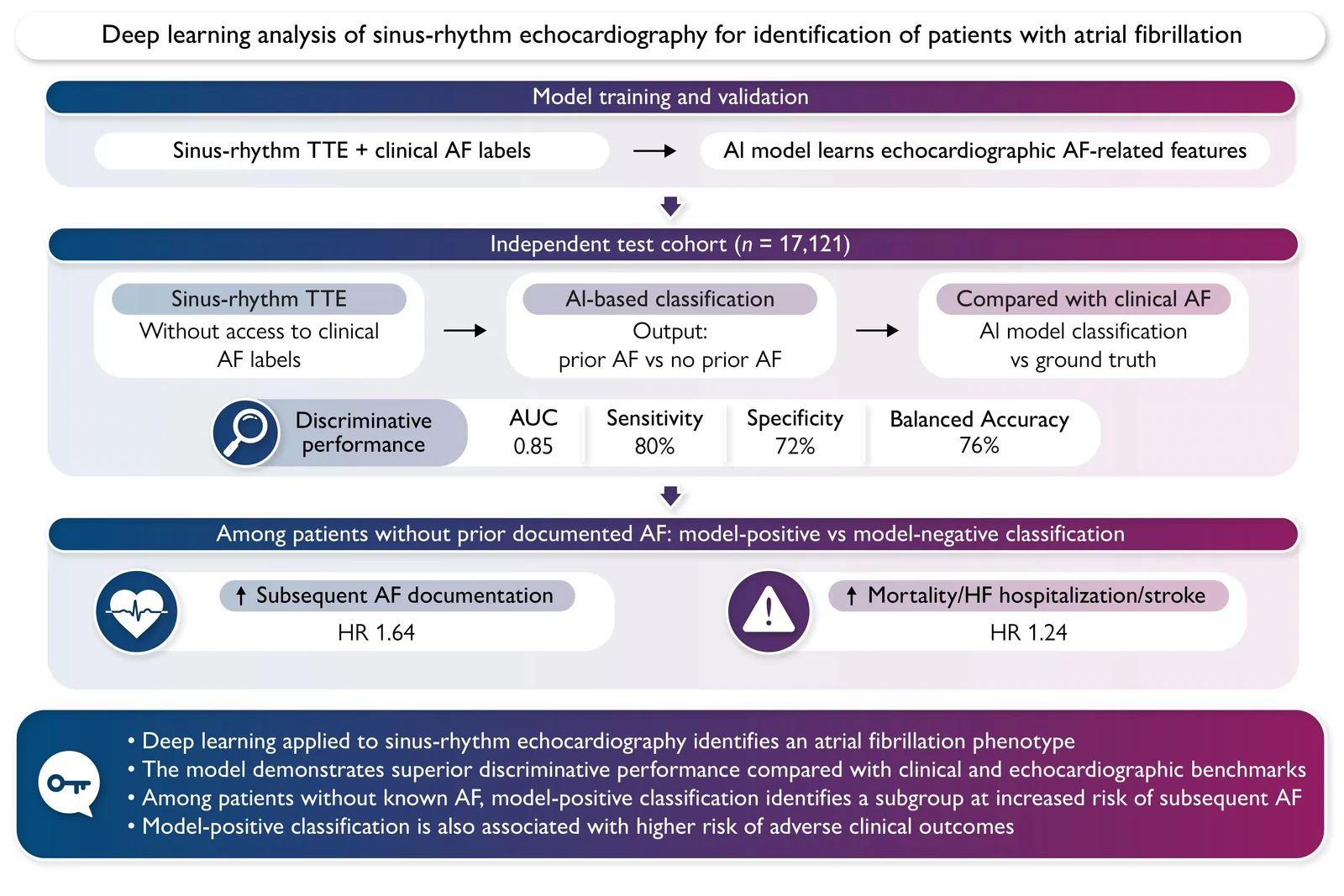
A deep learning model trained on sinus-rhythm transthoracic echocardiograms using clinical AF labels was evaluated in an independent test cohort (*n* = 17 121) without access to labels. Model classification was compared with clinical AF as the reference standard. The model demonstrated good discrimination (AUC 0.85). Among patients without prior AF, model-positive classification was associated with increased risk of subsequent AF documentation [adjusted hazard ratio (HR) 1.64] and adverse clinical outcomes (death, heart failure hospitalization, or stroke; adjusted HR 1.24).

What’s new?

- Deep learning applied to sinus-rhythm echocardiography discriminates between patients with and without clinically documented atrial fibrillation (AF).
- The model demonstrates superior discriminative performance for identifying AF compared with selected clinical risk scores and conventional echocardiographic measurements.
- Among patients without prior documented AF, model-positive classification is associated with a higher likelihood of subsequent AF documentation.
- Model-positive classification is also associated with increased risk of adverse clinical outcomes.

## Introduction

Atrial fibrillation (AF) is the most prevalent cardiac arrhythmia and is strongly associated with ischaemic stroke, heart failure (HF), and increased mortality.^1–3^ Diagnosing AF remains challenging, driven by both biological and healthcare-system factors. Many patients are asymptomatic, and paroxysmal AF can be missed by single time-point electrocardiogram (ECG) testing due to its intermittent nature. Extended rhythm monitoring improves detection but requires logistics, interpretation capacity, and sustained healthcare resources, limiting scalable implementation in routine care. Consequently, a meaningful proportion of AF remains clinically unrecognized; across cohorts, ∼10–20% of AF is undiagnosed, although estimates vary by population and intensity of rhythm surveillance. Importantly, many individuals in whom AF is not yet documented may have risk profiles that could warrant anticoagulation if AF were confirmed, underscoring the clinical value of more efficient pathways to rhythm confirmation.^4–6^

The under-recognition of AF has motivated guideline-supported screening and case-finding strategies tailored to age and risk factors.^7,8^ However, the key unmet need is not screening itself, but optimizing whom to monitor and how to allocate rhythm-testing resources to maximize diagnostic yield and clinical impact.

Recent advances in artificial intelligence (AI) offer new opportunities to enrich AF case-finding. AI applied to sinus-rhythm ECGs can identify electrical signatures associated with current or future AF.^9–12^ In parallel, AI applied to transthoracic echocardiography (TTE) has demonstrated diagnostic and prognostic utility across multiple cardiovascular conditions.^13,14^ Because AF is associated with patterns that can be reflected in echocardiographic anatomy and physiology,^15–18^ TTE may capture structural and functional features of atrial remodelling that are not reflected in ECG-derived electrical signatures, thereby providing complementary information to refine rhythm-monitoring strategies when applied opportunistically to already-indicated echocardiographic studies rather than as a primary screening tool.

Accordingly, we evaluated whether deep learning applied to sinus-rhythm TTE can discriminate patients with clinically documented AF, and whether model-positive classification is associated with subsequent AF documentation and adverse clinical outcomes.

## Methods

### Study design and data sources

We conducted a retrospective study of adult TTE examinations performed at Sheba Medical Center, a tertiary referral centre in Israel, between February 2009 and May 2021. Studies were acquired in the echocardiography laboratory across multiple ultrasound platforms and stored as digital cine loops and still frames according to routine clinical protocols. The study leveraged the SHEBAHEART registry, which integrates echocardiographic imaging data, echocardiography reports, and longitudinal electronic medical records.

Model development was performed by Aisap.ai in collaboration with Sheba Medical Center. The institutional review board approved the study with a waiver of informed consent, and all analyses were performed on de-identified data. The study was reported in accordance with the PRIME 2.0 (Reporting Guidelines for Clinical Artificial Intelligence Prediction Models) framework.^19^ A completed PRIME 2.0 checklist is provided in Supplementary material online, *Table S1*.

### Study population and cohort construction

We included adult TTE examinations acquired in sinus rhythm. Sinus rhythm was confirmed by a contemporaneous ECG obtained during the TTE visit. Examinations were excluded if AF was present on the ECG at the time of imaging or if the echocardiography report explicitly documented AF during the examination. To reduce confounding related to major interventions that may substantially alter cardiac structure or rhythm status, we excluded patients with prior valve replacement or repair, cardiac implantable electronic devices (pacemaker or defibrillator), heart transplantation, mechanical circulatory support, or invasive mechanical ventilation. Patients receiving chronic oral anticoagulation without documented AF were also excluded to minimize label ambiguity.

Initially, 157 638 eligible TTE examinations were identified during the study period. Following the exclusion of 28 841 examinations because of technical issues, 128 797 examinations remained available for AI analysis. Clinical exclusions further removed 5673 examinations acquired during AF, 10 159 examinations from patients whose first documented AF diagnosis occurred within three years after TTE, and 6707 examinations from patients receiving anticoagulation therapy without documented AF. Additional exclusions included 9775 examinations from patients with prior valve replacement or repair, 7702 from patients with pacemaker or defibrillator implantation, 2853 from patients with heart transplantation or circulatory support, and 242 obtained during invasive mechanical ventilation. The final dataset consisted of 85 686 TTE examinations (*Figure 1*).

**Figure 1:**
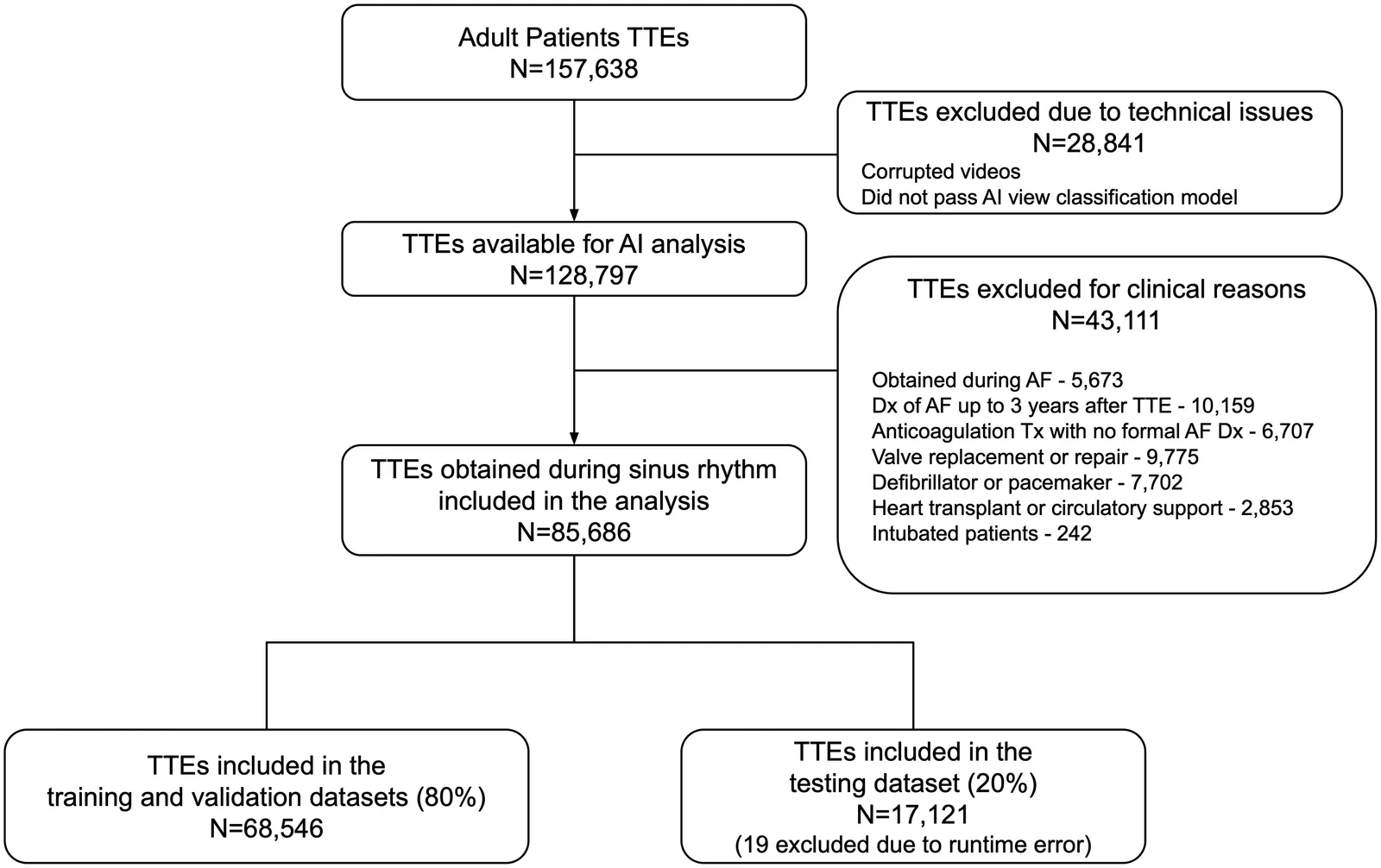
Cohort selection flowchart. Flow diagram showing inclusion and exclusion criteria for adult transthoracic echocardiography (TTE) examinations at Sheba Medical Center (2009–21). Of 157 638 TTEs, 28 841 were excluded due to technical issues and 43 111 for clinical reasons. The final dataset included 85 686 sinus rhythm TTEs: 68 546 for training/validation and 17 121 for testing.

The dataset was partitioned into training (60%), validation (20%), and independent hold-out test (20%) cohorts. The hold-out test set was constructed at the patient level such that each patient contributed a single index TTE examination and no patient appeared in both the test set and the training or validation sets. Multiple examinations from the same patient were permitted in the training and validation cohorts. Baseline clinical and echocardiographic characteristics of the training, validation, and independent test cohorts are provided in Supplementary material online, *Table S2*.

### Atrial fibrillation ascertainment and labelling

AF status was ascertained retrospectively from longitudinal clinical documentation available within the electronic medical record. AF documentation reflected routine clinical practice and was based on the earliest available evidence from any source, including International Classification of Diseases (ICD) diagnosis codes, 12-lead ECG reports, Holter monitor reports, and prior echocardiographic documentation of AF. Because these documentation sources were not mutually exclusive, AF classification could be supported by one or more sources of clinical evidence. ICD diagnoses at our institution are physician-assigned following review of available clinical records rather than derived solely from administrative billing data, and therefore often reflect AF diagnoses established either within or outside our institution. The earliest documented evidence from any source was considered the date of AF documentation. Examinations were labelled AF-positive if AF documentation preceded the TTE date. To reduce temporal misclassification due to delayed clinical recognition, patients whose first documented AF diagnosis occurred within three years after the qualifying TTE were excluded. All remaining examinations without prior AF documentation were labelled AF-negative.

### Echocardiographic data processing

Echocardiographic data consisted of B-mode cine loops acquired during routine clinical examinations. Prior to analysis, studies underwent automated technical validation. Examinations were excluded if cine files could not be processed because of corrupted or unsupported DICOM formats, missing required metadata (e.g. frame-rate information), missing image pixel data, or unsupported transfer syntaxes.

For eligible studies, videos underwent automated pre-processing. When ultrasound-region co-ordinates were available in the DICOM header, videos were cropped according to the ultrasound region, padded to a standardized 3:4 aspect ratio, and resized to 300 × 400 pixels. Additional pre-processing included contrast enhancement using contrast-limited adaptive histogram equalization (CLAHE) and image normalization. No explicit masking of video overlays, metadata, or ECG rhythm strips was performed.

Following pre-processing, echocardiographic views were automatically identified using an established EfficientNetV2^20^-based view-classification model trained on ∼12 000 manually labelled echocardiographic loops annotated by experienced sonographers. The classifier was developed independently of the present AF study, had been previously validated for automated echocardiographic view recognition, and is routinely used within a broader echocardiographic AI framework.^21^ Only loops meeting predefined view-classification confidence criteria were retained for downstream analysis.

### Deep learning model

The AF classification model analysed paired apical four-chamber (A4C) and parasternal long-axis (PLAX) greyscale echocardiographic views. These views were selected a priori because they provide complementary information from different imaging planes while remaining among the most routinely acquired and standardized echocardiographic views in clinical practice. Only examinations containing at least one qualifying A4C loop and one qualifying PLAX loop were analysed.

For each eligible examination, an instance-segmentation model was applied independently to each frame of the A4C and PLAX cine loops to delineate the relevant cardiac chambers. Frame-wise chamber area trajectories were then analysed to identify end-diastolic and end-systolic phases of the cardiac cycle. Ten frames were then deterministically sampled around these phases from each qualifying A4C loop and each qualifying PLAX loop, yielding paired A4C–PLAX inputs for model analysis. When multiple eligible A4C or PLAX loops were available within a study, all qualifying loops were retained.

The AF classification model combined an EfficientNetV2 convolutional backbone for spatial feature extraction with transformer-based components to integrate information across frames and capture temporal relationships. Features extracted from paired A4C and PLAX inputs were aggregated and passed to a classification head that generated a continuous probability representing the likelihood of prior clinically documented AF.

The model was trained using supervised learning with AF labels derived from the ascertainment strategy described above. Training optimization used a cross-entropy loss function.^22^ Data augmentation included temporal sampling variations, random cropping, and minor spatial perturbations to improve robustness and reduce overfitting.

During training, when multiple eligible A4C–PLAX loop combinations were available, one eligible loop pair was randomly selected at each epoch. During inference, predictions were generated across all eligible A4C–PLAX combinations (up to 25 loop pairs per examination), and the final examination-level probability was obtained by averaging predictions across combinations.

### Model evaluation and comparators

Model performance was evaluated in the independent hold-out test cohort by comparing model predictions against clinically documented AF. Performance metrics included area under the receiver operating characteristic curve (AUC), sensitivity, specificity, balanced accuracy, and *F*1 score. Classification thresholds were determined using the validation cohort and subsequently applied unchanged to the hold-out test cohort.

Performance was benchmarked against selected clinical and echocardiographic comparators, including the CHARGE-AF and CHA_2_DS_2_-VASc scores, left atrial anteroposterior diameter, and a parsimonious PLAX-based measurement set combined with age and sex (left atrial diameter, left ventricular end-diastolic diameter, left ventricular end-systolic diameter, interventricular septal thickness, and posterior wall thickness). CHARGE-AF was selected as an established clinical prediction model for incident AF.^23^ CHA_2_DS_2_-VASc was included as a widely available clinical score that incorporates several AF-associated risk factors and has been evaluated in prior studies of AF prediction and identification,^24,25^ although its primary clinical use is thrombo-embolic risk stratification in patients with known AF.^26^

Comparator variables were derived from structured clinical data and the index TTE report using information available at or prior to the TTE date, without access to future data.

### Model explainability

To support interpretability, we generated gradient-based saliency maps for representative examinations from the hold-out test cohort, including both model-positive and model-negative cases. Saliency maps were computed at the frame level and overlaid on the original images to visualize regions contributing most to the model output. The overlays were reviewed qualitatively by cardiologists with expertise in echocardiography to assess whether salient regions corresponded to recognizable cardiac structures and whether patterns were consistent across cases.

The analysis was descriptive and hypothesis-generating, did not inform model development, and saliency findings were interpreted cautiously without inferring mechanistic relationships.

### Clinical outcomes

Outcome analyses were performed in the hold-out test cohort (one index TTE per patient). Two clinical endpoints were evaluated to assess the prognostic associations of the model classifications. First, within the cohort without AF documented prior to the index TTE, we evaluated subsequent AF documentation during follow-up by comparing event rates between patients classified model-positive (ground truth–negative/model-positive) and those classified model-negative (ground truth–negative/model-negative). Second, we evaluated a composite clinical outcome of all-cause mortality, heart-failure hospitalization, or stroke, comparing risks across model–ground truth groups [true positives (TPs), false positives (FPs), true negatives (TNs), and false negatives (FNs)]. Each component of the composite outcome was also examined separately. These analyses were intended to determine whether model classifications, defined relative to clinically documented AF status, were associated with differential downstream risk profiles.

### Statistical analysis

Continuous variables were expressed as mean ± standard deviation if normally distributed, or as median with interquartile range (IQR) if skewed. Categorical variables were presented as frequencies and percentages. Between-group differences in continuous variables were assessed using one-way analysis of variance (ANOVA) or the Kruskal–Wallis test, as appropriate. The index date was defined as the date of the index TTE, and patients were followed until the occurrence of the relevant clinical event or until 1 October 2024.

Time-to-event analyses were performed using Kaplan–Meier estimates with log-rank tests. Cox proportional hazards regression models were used to estimate univariable and multivariable hazard ratios (HRs) with 95% confidence intervals (CIs). For subsequent AF documentation, analyses were restricted to patients without AF documented prior to the index TTE and compared outcomes between the model-positive and model-negative groups within this cohort. For the composite endpoint and its components, comparisons were made across model–ground truth groups (TPs, FPs, FNs) relative to TNs, recognizing that these categories are defined relative to clinically documented AF.

Multivariable models adjusted for clinically relevant covariates selected a priori, including age, sex, obesity (body mass index, BMI ≥30 kg/m^2^), hypertension, diabetes, HF, ischaemic heart disease, chronic lung disease, chronic kidney disease (estimated glomerular filtration rate <45 mL/min/1.73 m^2^), malignancy, dementia, prior stroke, left ventricular ejection fraction (LVEF) <40%, at least moderate aortic stenosis or mitral regurgitation, systolic pulmonary artery pressure ≥40 mmHg, right ventricular dysfunction, LV end-diastolic diameter, and left atrial diameter and area. Proportional hazards assumptions were assessed using Schoenfeld residuals. All analyses were performed in R version 4.3.3; a two-sided *P*-value <0.05 was considered statistically significant.

## Results

The final hold-out test cohort included 17 121 patients, of whom 3449 (20.1%) had prior documented AF before the index TTE. The median age was 66 years (IQR 54–75), and 9857 patients (57.6%) were male. Cardiac comorbidities included ischaemic heart disease in 6529 (38.1%) and HF in 1250 (7.3%). Valvular disease was prevalent, with aortic stenosis in 957 (5.6%), mitral regurgitation in 983 (5.7%), and tricuspid regurgitation in 726 (4.2%). Echocardiography demonstrated a mean LVEF of 55 ± 10.7%, a left atrial (LA) diameter of 38 ± 6 mm, and an LA end-systolic area of 20 ± 5 cm^2^; mean estimated systolic pulmonary artery pressure was 35 ± 11 mmHg (*Table 1*). During follow-up, the cohort accumulated 114 714 person-years for adverse outcomes (median 6.4 years; IQR 3.8–9.5), with 1049 (6.1%) HF hospitalizations, 720 (4.2%) strokes, and 5607 (32.7%) deaths. Among patients without prior documented AF (*n* = 13 672), follow-up for subsequent AF documentation totalled 114 375 person-years (median 8.2 years; IQR 5.4–11.1), and 1116 (8.2%) had subsequent AF documentation.

**Table 1:** Test set baseline clinical and echocardiographic characteristics

Characteristic	Entire Test set (*n* = 17 121)	True negative (*n* = 9840)	False negative (*n* = 700)	False positive (*n* = 3832)	True positive (*n* = 2749)
Age (years) (SD)	63.4 (15.9)	57.4 (15.4)	63.2 (14.3)	70.9 (12.7)	74.6 (10.9)
Male (%)	9857 (57.6)	5476 (55.7)	428 (61.1)	2330 (60.8)	1623 (59.0)
BMI (kg/m^2^) (SD)	27.1 (4.8)	26.7 (4.7)	27.0 (4.7)	27.5 (4.8)	27.7 (4.9)
Hypertension (%)	8030 (46.9)	3418 (34.7)	383 (54.7)	2230 (58.2)	1999 (72.7)
Diabetes mellitus (%)	4519 (26.4)	1889 (19.2)	193 (27.6)	1337 (34.9)	1100 (40.0)
Dyslipidaemia (%)	8483 (49.5)	4108 (41.7)	410 (58.6)	2159 (56.3)	1806 (65.7)
Smoking (%)	2399 (14.0)	1523 (15.5)	111 (15.9)	505 (13.2)	260 (9.5)
Ischemic heart disease (%)	6529 (38.1)	2803 (28.5)	271 (38.7)	1899 (49.6)	1556 (56.6)
Heart failure (%)	1250 (7.3)	252 (2.6)	54 (7.7)	381 (9.9)	563 (20.5)
COPD (%)	1296 (7.6)	461 (4.7)	76 (10.9)	362 (9.4)	397 (14.4)
Chronic kidney disease (%)	1520 (8.9)	326 (3.3)	51 (7.3)	553 (14.4)	590 (21.5)
Malignancy (%)	3003 (17.5)	1807 (18.4)	128 (18.3)	578 (15.1)	490 (17.8)
Prior CVA (%)	1947 (11.4)	808 (8.2)	104 (14.9)	508 (13.3)	527 (19.2)
Peripheral vascular disease (%)	1114 (6.5)	349 (3.5)	47 (6.7)	351 (9.2)	367 (13.4)
Dementia (%)	430 (2.5)	111 (1.1)	20 (2.9)	144 (3.8)	155 (5.6)
LVEF (%) (SD)	55.3 (10.7)	57.3 (8.6)	56.4 (9.7)	51.8 (13.1)	52.5 (12.2)
LVEDD (cm) (SD)	4.7 (0.6)	4.7 (0.5)	4.7 (0.6)	4.9 (0.7)	4.9 (0.7)
LVESD (cm) (SD)	3.1 (0.7)	2.9 (0.6)	3.0 (0.6)	3.3 (0.8)	3.3 (0.8)
LV interventricular septum thickness (cm) (SD)	1.1 (0.2)	1.0 (0.2)	1.1 (0.2)	1.1 (0.2)	1.1 (0.2)
LV posterior wall thickness (cm) (SD)	0.9 (0.2)	0.9 (0.2)	0.9 (0.2)	1.0 (0.2)	1.0 (0.2)
LV estimated mass index (g) (SD)	91.8 (27.9)	84.4 (24.1)	88.7 (25.7)	102 (29.2)	106 (29.7)
LA diameter (cm) (SD)	3.8 (0.6)	3.6 (0.5)	3.8 (0.5)	4.1 (0.6)	4.4 (0.6)
LA end-systolic area (cm^2^) (SD)	20.3 (5.1)	18.3 (3.9)	19.3 (4.3)	22.3 (4.9)	25.0 (5.3)
Mitral inflow *E*/*A* ratio (SD)	1.2 (0.6)	1.1 (0.4)	1.0 (0.4)	1.2 (0.6)	1.6 (0.8)
Tissue Doppler *E*/*e*′ ratio lateral (SD)	10.1 (4.9)	8.6 (3.7)	9.3 (4.2)	12.3 (5.5)	13.3 (5.8)
Systolic pulmonary arterial pressure (mmHg) (SD)	34.9 (11.1)	30.6 (7.5)	32.6 (8.8)	39.2 (11.9)	43.3 (12.8)
Estimated mean right atrial pressure (mmHg) (SD)	7.7 (3.4)	6.9 (2.7)	7.2 (2.8)	8.4 (3.7)	9.5 (4.2)
Aortic stenosis (%)	957 (5.6)	317 (3.2)	29 (4.1)	338 (8.8)	273 (9.9)
Mitral regurgitation (%)	983 (5.7)	196 (2.0)	28 (4.0)	362 (9.4)	397 (14.4)
Tricuspid regurgitation (%)	726 (4.2)	73 (0.7)	6 (0.9)	206 (5.4)	441 (16.0)

Baseline clinical and echocardiographic characteristics of the hold-out test cohort, stratified by model prediction and ground-truth classification.

BMI, body mass index; COPD, chronic obstructive pulmonary disease; LA, left atrium; LV, left ventricle; LVEF, left ventricular ejection fraction; LVESD, left ventricular end-systolic diameter.

AF ascertainment was supported by multiple clinical documentation sources in most AF-positive patients. Details regarding overlap between ascertainment sources, the number of supporting documentation sources per patient, and sensitivity analyses according to the first documented AF ascertainment pathway are presented in Supplementary material online, *Figure S1*.

The model achieved an AUC of 0.85 with sensitivity 0.80, specificity 0.72, accuracy 0.74 (balanced accuracy 0.76), and *F*1 score 0.55 (*Table 2*). At the selected operating point, the model classified 6581 patients (38.4%) as model-positive; 3832 of these had no prior documented AF [i.e. ground truth–negative/model-positive (FP)]. Performance was compared with selected clinical and echocardiographic comparators, all of which demonstrated lower AUC values: 0.77 for PLAX-based measurements, 0.72 for left atrial size, 0.71 for the CHARGE-AF score, and 0.70 for the CHA_2_DS_2_-VASc score (*Figure 2*).

**Table 2:** Model performance for identifying patients with paroxysmal atrial fibrillation in the test set

AUC	Sensitivity	Specificity	Accuracy	*F*1 score
0.85	0.8	0.72	0.74	0.55

Diagnostic performance of the model using apical four-chamber and parasternal long-axis views acquired during sinus rhythm, evaluated against clinically documented atrial fibrillation (AF). Metrics include AUC, sensitivity, specificity, accuracy, and *F*1 score.

**Figure 2:**
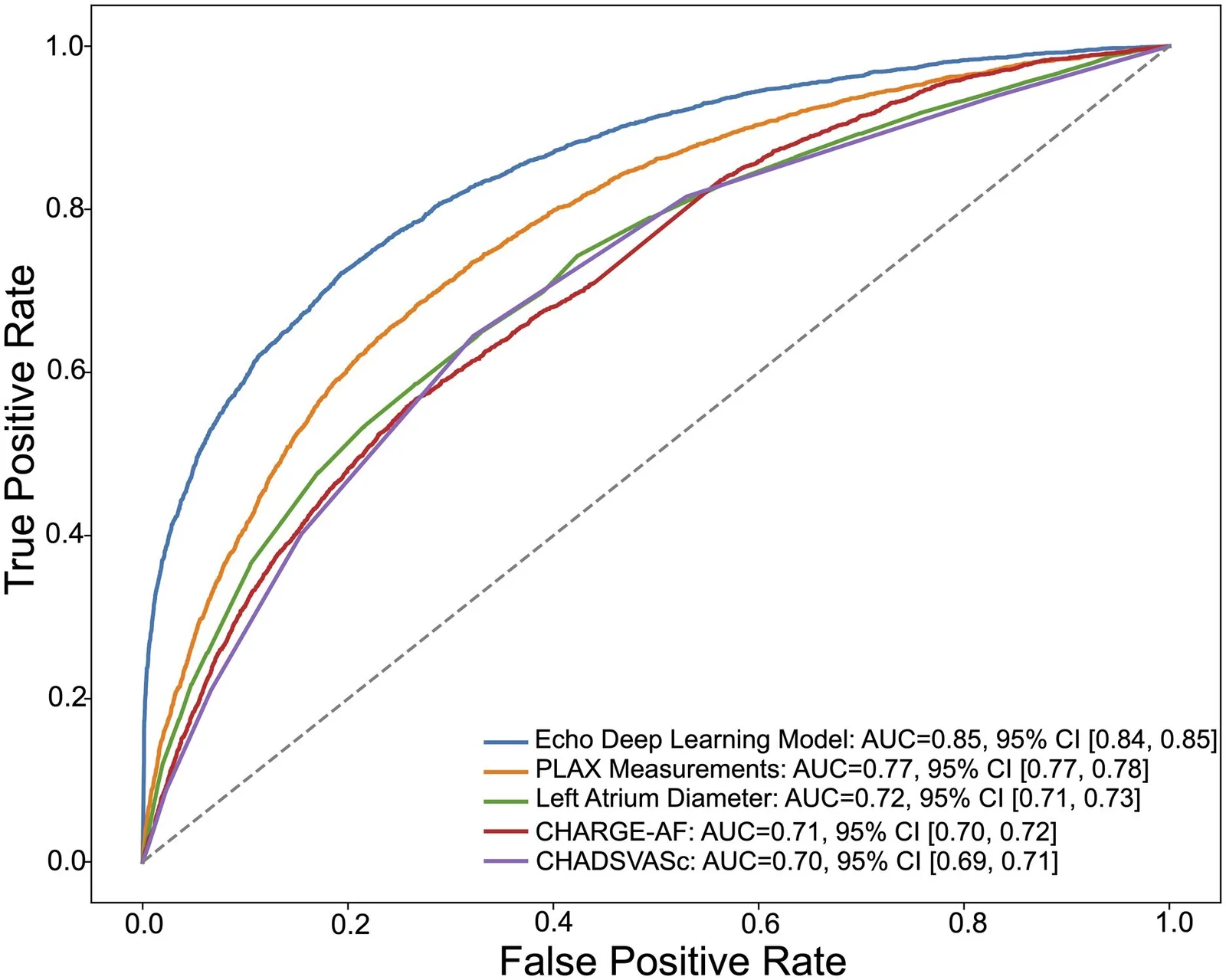
Comparative discriminative performance of echocardiography-based deep learning and conventional tools for identifying patients with atrial fibrillation. ROC curves comparing the discriminative performance of the deep learning model (AUC of 0.85; 95% CI 0.84–0.85) with PLAX-based measurements (0.77), LA diameter (0.72), CHARGE-AF (0.71), and CHA_2_DS_2_-VASc (0.70).

In exploratory interpretability analyses, saliency maps were generated for selected examinations from the hold-out test set, focusing on model-positive cases with high predicted probabilities and including model-negative cases for comparison. Saliency signals were observed in regions encompassing the mitral and aortic valves, left atrium (LA), and interatrial septum (*Figure 3*). These attribution maps were intended for descriptive interpretation only and should not be considered mechanistic evidence.

**Figure 3:**
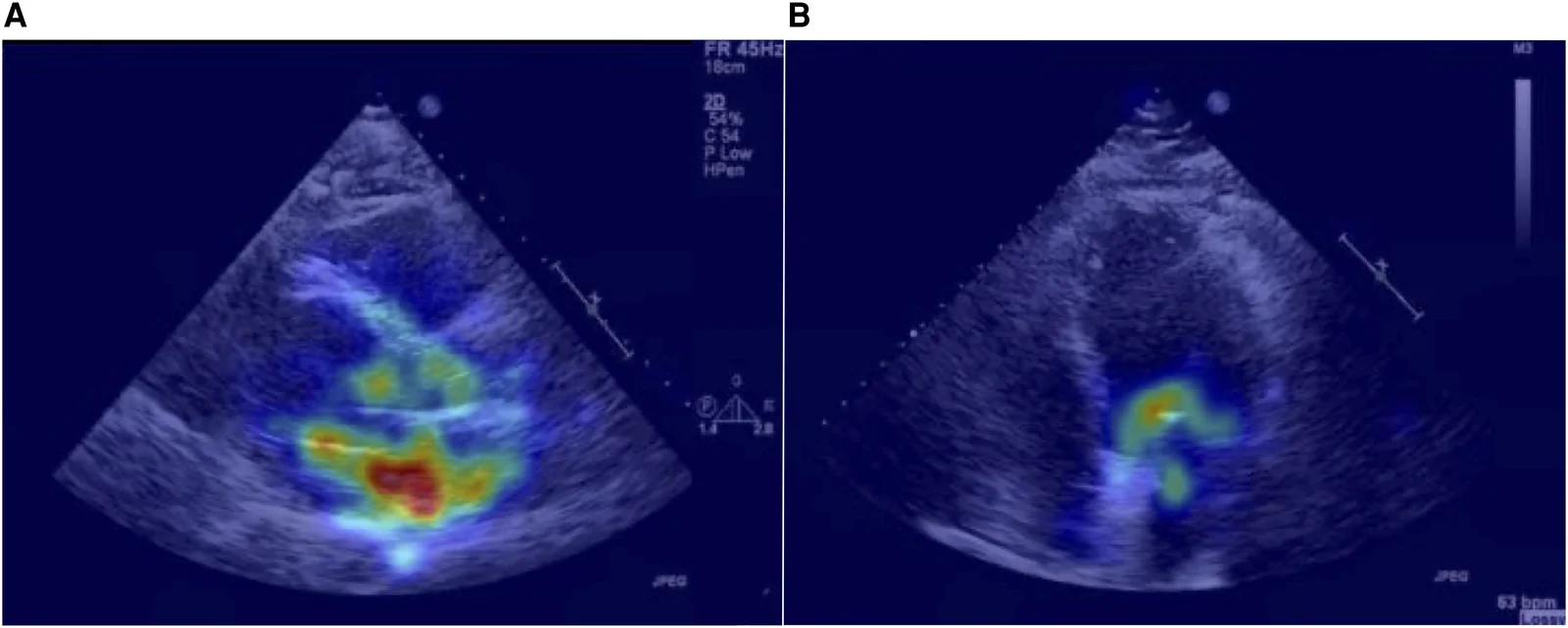
Saliency map analysis illustrating regions associated with AI-based classification of paroxysmal atrial fibrillation. Gradient-based saliency maps from PLAX (*A*) and A4C (*B*) views. The model emphasized the mitral and aortic valves, left atrium, and interatrial septum, structures previously associated with AF-related remodelling.

Within the cohort without prior documented AF (*n* = 13 672), patients classified as ground truth–negative/model-positive (FP; *n* = 3832) had higher rates of subsequent AF documentation than ground truth–negative/model-negative patients (TN; *n* = 9840): 602 (15.7%) vs. 514 (5.2%). The corresponding unadjusted HR was 3.41 (95% CI 3.03–3.83; *P* < 0.001), and the adjusted HR was 1.64 (95% CI 1.33–2.04; *P* < 0.001) (*Table 3*). Kaplan–Meier analysis demonstrated cumulative incidence of subsequent AF documentation of 17.1% ± 0.7% vs. 5.8% ± 0.25% at 8.2 years in FP and TN patients, respectively (*Figure 4*).

**Table 3:** Subsequent atrial fibrillation documentation among ground truth–negative patients by model classification

	True negative (*n* = 9840)	False positive (*n* = 3832)
Subsequent AF documentation, *n* (%)	514 (5.2)	602 (15.7)
Unadjusted HR (95% CI; *P*-value)	Reference	3.41 (3.03–3.83; *P* < 0.001)
Adjusted HR (95% CI; *P*-value)	Reference	1.64 (1.33–2.04; *P* < 0.001)

Incidence and hazard ratios (HRs) with 95% confidence intervals and *P*-values for subsequent atrial fibrillation (AF) documentation among patients without prior AF, stratified by model classification.

**Figure 4:**
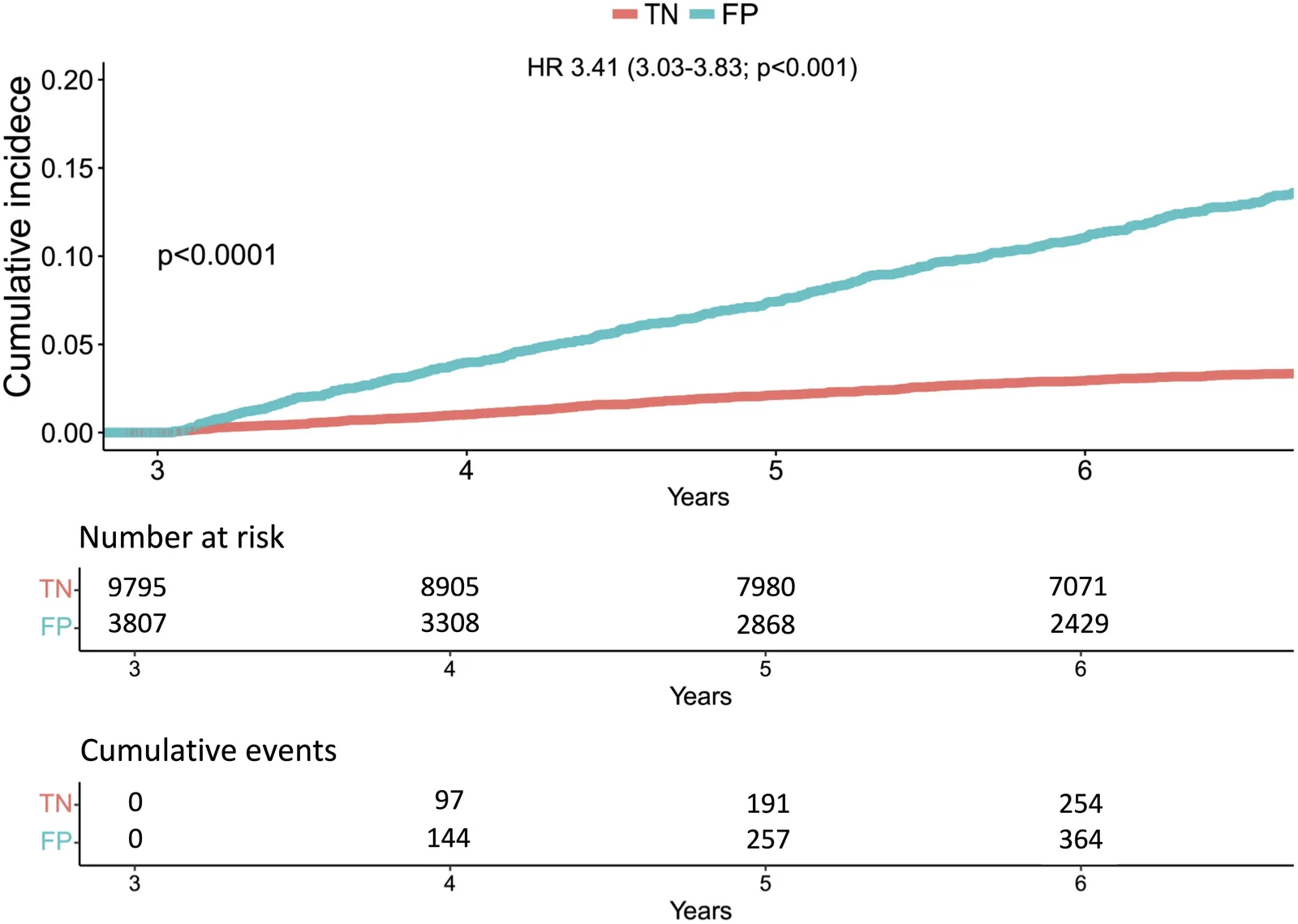
Cumulative incidence of subsequent atrial fibrillation in patients without prior AF diagnosis. Kaplan–Meier curves showing cumulative incidence of subsequent AF in patients without prior AF. Model-positive (FP) patients demonstrated a higher incidence of subsequent AF documentation compared with model-negative (TN) patients (unadjusted HR 3.41; 95% CI 3.03–3.83; *P* < 0.001).

A composite endpoint of all-cause mortality, HF hospitalization, or stroke was evaluated across model–ground truth groups. Compared with TNs, the FP group demonstrated higher risk (adjusted HR 1.24; 95% CI 1.13–1.35; *P* < 0.001). Kaplan–Meier analysis demonstrated cumulative incidence of the composite outcome of 29.0% ± 1.8%, 43.3% ± 0.86%, 18.6% ± 0.4%, and 58.5% ± 1.0% among FN, FP, TN, and TP groups, respectively, at 6.4 years (*Figure 5*). Significant associations were also observed for all-cause mortality (HR 1.27; 95% CI 1.15–1.39; *P* < 0.001) and HF hospitalization (HR 1.79; 95% CI 1.39–2.31; *P* < 0.001), whereas stroke was significant in univariable analysis but not after multivariable adjustment (*Tables 4–6*).

**Figure 5:**
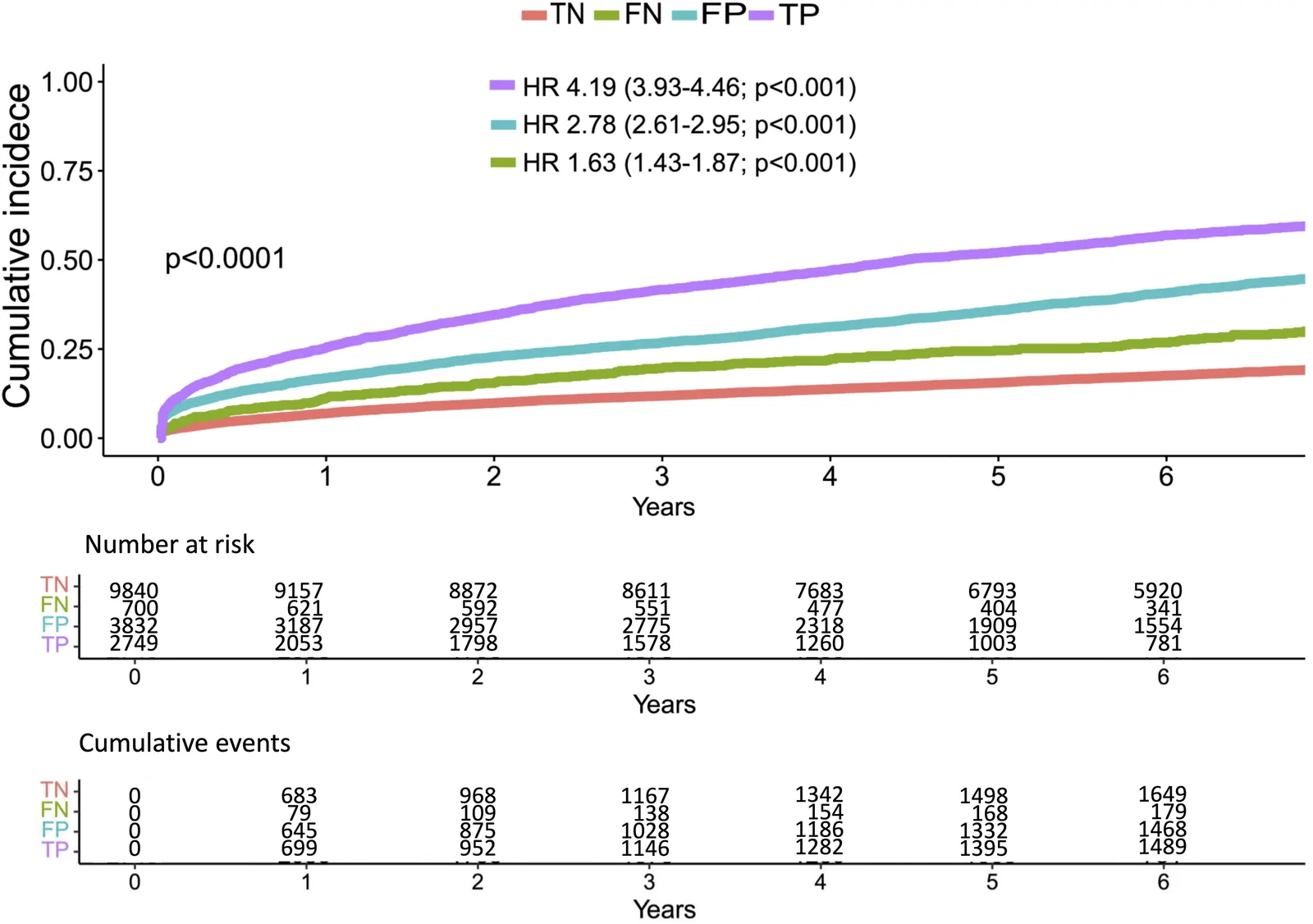
Cumulative incidence of composite outcome by model-based atrial fibrillation classification. Cumulative incidence of a composite outcome (mortality, heart failure hospitalization, or stroke) by classification group. FP patients demonstrated higher unadjusted risk compared with TN (HR 2.78; 95% CI 2.61–2.95). Elevated risks were also observed in FN and TP groups relative to TN.

**Table 4:** Incidence of adverse clinical outcomes by atrial fibrillation classification group

Outcome	Entire test set (*n* = 17 121)	True negative (*n* = 9840)	False negative (*n* = 700)	False positive (*n* = 3832)	True positive (*n* = 2749)
Composite outcome (all-cause mortality, heart failure hospitalization, or stroke) (%)	6103 (35.6)	2272 (23.0)	231 (33.0)	1901 (49.6)	1699 (61.8)
All-cause mortality (%)	5607 (32.7)	2021 (20.5)	211 (30.1)	1770 (46.2)	1605 (58.3)
Heart failure hospitalization (%)	1049 (6.1)	182 (1.8)	28 (4.0)	358 (9.3)	481 (17.5)
Stroke (%)	720 (4.2)	341 (3.5)	35 (5.0)	191 (5.0)	153 (5.5)

Event rates for the composite outcome (all-cause mortality, heart failure hospitalization, or stroke) and its individual components, stratified by classification group.

**Table 5:** Unadjusted hazard ratios by atrial fibrillation classification group

Outcome	False negative	False positive	True positive
Composite outcome (all-cause mortality, heart failure hospitalization, or stroke)	1.63 (1.43–1.87; *P* < 0.001)	2.78 (2.61–2.95; *P* < 0.001)	4.19 (3.93–4.46; *P* < 0.001)
All-cause mortality	1.68 (1.46–1.94; *P* < 0.001)	2.88 (2.70–3.07; *P* < 0.001)	4.37 (4.09–4.66; *P* < 0.001)
Heart failure hospitalization	2.33 (1.57–3.47; *P* < 0.001)	5.92 (4.95–7.08; *P* < 0.001)	12.70 (10.70–15.10; *P* < 0.001)
Stroke	1.54 (1.09–2.18; *P* = 0.02)	1.62 (1.35–1.93; *P* < 0.001)	1.97 (1.63–2.39; *P* < 0.001)

Unadjusted hazard ratios (HRs) with 95% confidence intervals and *P*-values for adverse outcomes across groups, using true negative (TN) as reference.

**Table 6:** Adjusted hazard ratios by atrial fibrillation classification group

Outcome	False negative	False positive	True positive
Composite outcome (all-cause mortality, heart failure hospitalization, or stroke)	1.11 (0.94–1.32; *P* = 0.20)	1.24 (1.13–1.35; *P* < 0.001)	1.37 (1.23–1.52; *P* < 0.001)
All-cause mortality	1.15 (0.95–1.38; *P* = 0.14)	1.27 (1.15–1.39; *P* < 0.001)	1.45 (1.30–1.62; *P* < 0.001)
Heart failure hospitalization	1.39 (0.85–2.27; *P* = 0.20)	1.79 (1.39–2.31; *P* < 0.001)	2.35 (1.78–3.09; *P* < 0.001)
Stroke	1.09 (0.72–1.64; *P* = 0.70)	0.96 (0.75–1.22; *P* = 0.70)	0.86 (0.64–1.16; *P* = 0.30)

Multivariable-adjusted hazard ratios (HRs) with 95% confidence intervals and *P*-values for adverse outcomes across groups, using true negative (TN) as reference.

## Discussion

This study demonstrates that a deep learning model applied to TTE acquired during sinus rhythm can discriminate between patients with and without clinically documented AF, despite the absence of arrhythmia at the time of imaging. The model demonstrated higher discriminative performance than selected clinical comparators and conventional echocardiographic measurements. Leveraging routinely acquired echocardiographic data, it identified a phenotype associated with AF and with increased likelihood of subsequent AF documentation and adverse clinical outcomes. These findings should be interpreted as reflecting an imaging-derived signal associated with AF-related disease, rather than direct detection of occult arrhythmia or prediction of future AF events.

Echocardiography is routinely performed in patient populations in whom AF detection carries diagnostic and therapeutic implications.^27–29^ Because AF is associated with structural and functional cardiac remodelling, echocardiography may capture disease-related changes even when patients are in sinus rhythm at the time of imaging. While AI-enabled ECG models have demonstrated the ability to identify AF-related electrical signatures, echocardiography is not intended to replace ECG-based approaches, but rather to provide complementary information by capturing structural and functional manifestations of the same disease process. Accordingly, TTE examinations already performed as part of routine clinical care may offer an opportunity for AF case-finding without additional testing.

Traditional echocardiographic approaches to AF risk assessment rely on discrete measurements such as left atrial diameter, volume, or Doppler indices, which capture only selected components of this remodelling and are subject to variability in acquisition and interpretation.^30–36^ Although multivariable models based on these parameters may partially address these limitations, they require standardized measurements and structured reporting, limiting scalability. Beyond these practical constraints, conventional parameters provide only a simplified representation of the underlying imaging data. Much of this signal is embedded in complex spatiotemporal echocardiographic data and is not captured by predefined measurements. In this context, deep learning enables direct analysis of raw echocardiographic video, integrating spatial and temporal features without pre-specified assumptions. This approach may uncover clinically relevant information that remains inaccessible to conventional analysis, broadening the diagnostic potential of echocardiography beyond overt structural abnormalities and helping refine AF case-finding strategies.

Examination of the saliency maps together with the clinical and echocardiographic characteristics of model-positive patients provides additional insight into the imaging phenotype associated with model classification. Compared with model-negative patients, model-positive patients tended to be older and were characterized by a higher burden of cardiovascular comorbidities together with more advanced structural and functional cardiac remodelling, including greater left atrial enlargement, higher filling and pulmonary pressures, greater left ventricular mass, more frequent valvular abnormalities, and a modest reduction in left ventricular systolic function. These findings are broadly consistent with the saliency maps, which predominantly highlighted the LA, mitral and aortic valves, and interatrial septum. Although these observations do not establish the specific imaging features driving model predictions, they suggest that the model captures a broader phenotype of cardiac remodelling that is common in AF but also overlaps with other cardiovascular conditions. Accordingly, the biological signal identified by the model likely reflects shared remodelling pathways rather than disease-specific imaging markers, and the precise mechanisms underlying model predictions remain to be fully elucidated.

Extending these observations to clinical outcomes, among patients without prior documented AF, model-positive classification was associated with nearly three-fold higher rates of subsequent AF documentation and modestly increased risks of mortality, HF hospitalization, and stroke after adjustment for confounders. From a clinical and electrophysiological standpoint, these associations may have implications across a spectrum of potential interventions. Patients identified in this manner may be candidates for general preventive strategies such as weight reduction, blood pressure optimization, alcohol moderation, and screening for sleep apnoea—low-risk interventions known to reduce AF incidence. Consideration may also be given to more intensive rhythm monitoring, ranging from standard ECG to extended ambulatory monitoring or implantable loop recorders, as well as to potential therapeutic strategies such as anticoagulation or rhythm-control therapy in the absence of confirmed AF. In selected populations at therapeutic decision thresholds, such as patients with embolic stroke of undetermined source (ESUS), such approaches may be of particular interest. However, given the lack of benefit of empiric anticoagulation in unselected ESUS populations,^37–40^ any model-informed strategy would require careful prospective evaluation. All such applications should be considered hypothesis-generating and require prospective validation before clinical implementation.

This study builds on prior work by Yuan *et al*.,^41^ who applied deep learning to TTEs from the PLAX view to predict AF within 90 days, achieving moderate performance (AUC 0.74 in internal validation). Our approach extends this concept by incorporating two complementary standard views—PLAX and A4C—and demonstrates improved discriminative performance. Moreover, we link model-based classifications to longitudinal clinical outcomes, enhancing clinical relevance and demonstrating associations with subsequent events.

This study has several notable strengths. The large, consecutive cohort included diverse cardiovascular conditions encountered in routine clinical practice. The model operated fully automatically, without additional imaging or manual input, supporting scalability. Importantly, the model was designed to identify imaging features associated with established AF rather than to predict future events, consistent with current clinical practice in which interventions such as anticoagulation are based on documented AF.

Nevertheless, several limitations should be acknowledged. This was a single-centre study conducted at a tertiary referral institution without external validation, limiting generalizability across populations, clinical settings, and imaging environments. Although examinations were acquired across multiple ultrasound platforms, subgroup analyses according to ultrasound vendor, system model, and acquisition settings were not performed. In addition, the study population was restricted to patients referred for clinically indicated echocardiography, and exclusion of patients with prior cardiac interventions may further limit applicability to broader and more complex populations. AF ascertainment relied on routine clinical documentation rather than systematic rhythm monitoring and was therefore susceptible to under-detection and temporal misclassification. Direct comparison with established alternative approaches was also limited, as an ECG-based AI comparator was not available and left atrial volume index and left atrial strain measurements were not routinely collected. ECG rhythm strips and other image overlays were not systematically removed from model inputs, and although several factors reduce the likelihood of clinically meaningful information leakage—including the use of a non-standardized single-lead sinus-rhythm strip, random cropping during training, and saliency maps that predominantly highlighted cardiac structures rather than ECG-containing regions—residual leakage cannot be completely excluded. Finally, model interpretability remains inherently limited, as saliency mapping provides qualitative rather than mechanistic insight into the imaging features underlying model predictions.

Future work should prioritize external validation across diverse populations, clinical settings, and imaging platforms. Evaluating model performance in point-of-care ultrasound (POCUS) or abbreviated studies acquired by less experienced operators will be important to assess real-world feasibility and robustness in lower-information imaging environments. Conversely, future studies should explore whether broader integration of echocardiographic data, including additional views, Doppler information, or complete TTE examinations, can further improve performance and clinical discrimination. Prospective studies are needed to determine clinical utility, particularly whether management strategies informed by model-based classification translate into improved outcomes. Even with demonstrated clinical effectiveness, practical considerations remain, including standardization of image acquisition, definition of classification thresholds, integration into diagnostic and therapeutic pathways, and reimbursement policies, all of which will be essential for successful clinical implementation.

### Conclusions and clinical implications

In conclusion, deep learning applied to TEE acquired during sinus rhythm can discriminate between patients with and without prior clinically documented AF. Model-positive classification was associated with subsequent AF documentation and an increased risk of adverse clinical outcomes, supporting the potential clinical utility of this approach.

These findings highlight the ability of routinely acquired echocardiographic data to capture an imaging phenotype associated with clinically documented AF and subsequent adverse outcomes. Further studies, including external validation and prospective evaluation, are needed to determine generalizability and clinical utility. In particular, randomized studies will be required to assess whether management strategies informed by model-based classification improve outcomes beyond current guideline-directed care.

## Supplementary Material

ztag168_Supplementary_Data

## Data Availability

The imaging data are housed at the Sheba Medical Center echocardiography laboratory. Due to third-party contractual agreements, these data cannot be publicly shared. Requests for onsite access to the original data may be directed to the corresponding author, subject to appropriate data-use agreements. The model code is proprietary intellectual property of Aisap.ai and is not publicly available. However, access to the software for reproducibility purposes may be requested from the sponsor.

## Abbreviations

AF: Atrial fibrillation
AI: Artificial intelligence
ANOVA: Analysis of variance
AUC: Area under the receiver operating characteristic curve
A4C: Apical four-chamber view
BMI: Body mass index
CI: Confidence interval
COPD: Chronic obstructive pulmonary disease
CLAHE: Contrast-limited adaptive histogram equalization
DNN: Deep neural network
ECG: Electrocardiogram
ESUS: Embolic stroke of undetermined source
FN: False negatives
FP: False positives
HF: Heart failure
HR: Hazard ratio
ICD: International Classification of Diseases
IQR: Interquartile range
LA: Left atrium
LV: Left ventricle
LVEDD: Left ventricular end-diastolic diameter
LVEF: Left ventricular ejection fraction
LVESD: Left ventricular end-systolic diameter
PLAX: Parasternal long-axis view
POCUS: Point-of-care ultrasound
TN: True negatives
TP: True positives
TTE: Transthoracic echocardiography
