# Supplementary material for: Deep learning analysis of sinus-rhythm echocardiography for the identification of patients with atrial fibrillation
Source: Eur Heart J Digit Health. 2026 Oct 21;7(9):ztag168. doi: 10.1093/ehjdh/ztag168 (PMC13714549; doi:10.1093/ehjdh/ztag168)
Supplement: Supplementary file 1 [file ztag168_supplementary_data.docx]

**Supplementary Table S1. PRIME 2.0 checklist and manuscript cross-reference**

Completed PRIME 2.0 reporting checklist indicating where each reporting item is addressed in the revised manuscript

| **Section** | **Checklist item** | **Manuscript section(s)** | **Table/Figure** |
| --- | --- | --- | --- |
| **1** | **Designing an AI Study in Cardiovascular Imaging** |  |  |
| *1.1* | *Appropriateness of applying AI* |  |  |
| a | Describe the need for applying AI | Introduction |  |
| b | Determine the appropriateness of applying AI | Discussion |  |
| *1.2* | *Study objectives, input data type, and prediction target* |  |  |
| a | Explain the AI task and the likely deployment context | Introduction; Discussion |  |
| b | Describe the input data, number of training/test examples | Methods - Study Design and Data Sources; Study Population and Cohort Construction | Figure 1; Supplementary Table S2 |
| c | Specify model supervision type | Methods - AF Ascertainment and Labeling; Deep Learning Model |  |
| d | Describe the nature of the model’s output and what it represents | Methods - Deep Learning Model; Clinical Outcomes |  |
| *1.3* | *Design of the AI study* |  |  |
| a | Describe the study design | Methods - Study Design and Data Sources |  |
| b | Describe data origin | Methods - Study Design and Data Sources |  |
| c | Describe if impact analysis was included | Not applicable |  |
| **2** | **Data Format and Preprocessing** |  |  |
| *2.1* | *Clinical characteristics of the study cohort* |  |  |
| a | Present the age, sex, and race/ethnicity distributions of the cohort(s) | Results | Table 1; Supplementary Table S2 |
| b | Summarize key clinical, treatment, and imaging characteristics of the cohort(s) | Results | Table 1; Supplementary Table S2 |
| c | Compare summary statistics of cases and controls | Results | Table 1; Supplementary Table S2 |
| **3** | **Selection of AI Methods and Applications** |  |  |
| *3.1* | *Selecting appropriate AI methods and applications: Clearly define data composition (structured/unstructured)* | Methods - Echocardiographic Data Processing; Deep Learning Model |  |
| *3.2, 3.3, 3.4* | *Training strategies: Describe the AI method/application used, with rationale for clinical task fit* | Methods - Deep Learning Model; Discussion |  |
| **4** | **Model Assessment** |  |  |
| *4.1* | *Importance of model assessment: Describe the evaluation approach and how it addresses the clinical question* | Methods - Model Evaluation and Comparators; Statistical Analysis; Results | Tables 2-4; Figures 2, 4, 5 |
| *4.2* | *Technical performance metrics* |  |  |
| a | Report relevant performance metrics (F1-score, IoU, BLEU, etc.) | Results | Table 2;  Figure 2 |
| b | Justify metric selection based on task characteristics | Methods - Model Evaluation and Comparators | Table 2;  Figure 2 |
| c | Describe manual annotation process, reference-standard prep, and observer variability | Methods - AF Ascertainment and Labeling | Supplementary Figure S1 |
| **5** | **Clinical Evaluation** |  |  |
| *5.1* | *Importance of clinical evaluation: Describe potential clinical impact of misclassification* | Discussion | Tables 3-4; Figures 4-5 |
| *5.2* | *Clinical validation: Provide evidence of clinical validation* | Results | Tables 3-4; Figures 4-5 |
| **6** | **Best Practices for Replicability** |  |  |
| *6.1* | *Importance of transparency and open science principles* |  |  |
| a | Ensure data sharing follows FAIR principles | Data Availability Statement |  |
| b | Report on training data representativeness | Methods - Study Population and Cohort Construction | Supplementary Table S2 |
| **7** | **Reporting of Limitations, Biases, and Alternatives** |  |  |
| *7.1* | *Acknowledging study and model limitations* |  |  |
| a | Discuss key limitations (data, methodology, generalizability) | Discussion - Limitations |  |
| b | Report sensitivity analyses and model-specific issues | Results; Discussion - Limitations | Supplementary Figure S1 |
| *7.2* | *Discussing study strengths: Articulate strengths (methodological rigor, dataset quality, innovation, clinical relevance)* | Discussion - Strengths |  |

**Supplementary Table S2. Baseline Clinical and Echocardiographic Characteristics of the Training, Validation, and Independent Test Cohorts**

Clinical and echocardiographic characteristics of patients included in the training (n=55,670), validation (n=12,876), and independent test (n=17,121) cohorts used for model development and evaluation.

| **Characteristic** | **Training Set**  (n=55,670) | **Validation Set**  (n=12,876) | **Test set**  (n=17,121) |
| --- | --- | --- | --- |
| Age (years) (SD) | 60.8  (17.7) | 60.5  (17.7) | 63.4  (15.9) |
| Male (%) | 31,155  (56.0) | 7,171  (55.7) | 9,857  (57.6) |
| BMI (kg/m^2^) (SD) | 27.0  (4.9) | 26.8  (4.9) | 27.1  (4.8) |
| Hypertension (%) | 18,541  (33.3) | 4,228  (32.8) | 8,030  (46.9) |
| Diabetes Mellitus (%) | 9,948  (17.9) | 2,249  (17.5) | 4,519  (26.9) |
| Dyslipidemia (%) | 19,949  (35.8) | 4,638  (36.0) | 8,483  (49.5) |
| Smoking (%) | 7,427  (13.3) | 1,798  (14.0) | 2,399  (14.0) |
| Ischemic Heart Disease (%) | 14,523  (26.1) | 3,355  (26.1) | 6,529  (38.1) |
| Heart Failure (%) | 3,730  (6.7) | 837  (6.5) | 1,250  (7.3) |
| COPD (%) | 2,195  (3.9) | 486  (3.8) | 1,296  (7.6) |
| Chronic Kidney Disease (%) | 4,170  (7.5) | 949  (7.4) | 1,520  (8.9) |
| Malignancy (%) | 6,637  (11.9) | 1,619  (12.6) | 3,003  (17.5) |
| Prior CVA (%) | 5,720  (10.3) | 1,321  (10.3) | 1,947  (11.4) |
| Peripheral Vascular Disease (%) | 1,731  (3.1) | 410  (3.2) | 1,114  (6.5) |
| Dementia (%) | 445  (0.8) | 114  (0.9) | 430  (2.5) |
| LVEF (%) (SD) | 56.4  (9.7) | 56.3  (9.7) | 55.3  (10.7) |
| LVEDD (cm) (SD) | 4.7  (0.6) | 4.7  (0.6) | 4.7  (0.6) |
| LVESD (cm) (SD) | 3.0  (0.7) | 3.0  (0.7) | 3.1  (0.7) |
| LV interventricular septum thickness (cm) (SD) | 1.0  (0.2) | 1.0  (0.2) | 1.1  (0.2) |
| LV posterior wall thickness (cm) (SD) | 0.9  (0.2) | 0.9  (0.2) | 0.9  (0.2) |
| LV estimated mass index (g) (SD) | 88.9  (27.2) | 88.9  (27.2) | 91.8  (27.9) |
| LA diameter (cm) (SD) | 3.8  (0.6) | 3.8  (0.6) | 3.8  (0.6) |
| LA end systolic area (cm^2^) (SD) | 19.8  (5.1) | 19.7  (5.0) | 20.3  (5.1) |
| Mitral Inflow E/A Ratio (SD) | 1.2  (0.6) | 1.2  (0.6) | 1.2  (0.6) |
| Tissue Doppler E/e’ Ratio Lateral (SD) | 9.6  (4.7) | 9.6  (4.7) | 10.1  (4.9) |
| Systolic Pulmonary Arterial Pressure (mmHg) (SD) | 33.9  (10.7) | 33.7  (10.4) | 34.9  (11.1) |
| Estimated mean right atrial pressure (mmHg) (SD) | 7.5  (3.4) | 7.5  (3.3) | 7.7  (3.4) |
| Aortic Stenosis (%) | 2,904  (5.2) | 625  (4.9) | 957  (5.6) |
| Mitral Regurgitation (%) | 2,737  (4.9) | 619  (4.8) | 983  (5.7) |
| Tricuspid Regurgitation (%) | 2,019  (3.6) | 443  (3.4) | 726  (4.2) |

**Supplementary Figure S1. AF ascertainment sources and sensitivity analyses**

(A) Overlap between AF ascertainment sources. (B) Distribution of AF-positive patients according to the number of documentation sources supporting AF ascertainment. (C) Sensitivity analysis according to the first documented AF ascertainment source. Model performance remained consistent across ascertainment pathways. Interpretation of the Holter subgroup is limited by the small sample size (n = 15).

**A. Overlap between AF ascertainment sources**

**B. Distribution of AF-positive patients according to the number of AF ascertainment sources**

| **Number of documentation sources** | **AF-positive patients, n (%)** |
| --- | --- |
| 1 | 946 (27.4) |
| 2 | 1,391 (40.3) |
| 3 | 863 (25.0) |
| 4 | 249 (7.2) |

**C. Sensitivity analysis according to the first documented AF ascertainment source**

| **First documented AF ascertainment source** | **AF-positive patients, n (%)** | **AUC** | **Sensitivity** | **Specificity** | **Accuracy** | **F1 score** |
| --- | --- | --- | --- | --- | --- | --- |
| Overall | 3,449 (100.0) | 0.85 | 0.80 | 0.72 | 0.74 | 0.55 |
| ICD diagnosis code | 2,178 (63.1) | 0.85 | 0.74 | 0.81 | 0.79 | 0.50 |
| ECG | 1,100 (31.9) | 0.83 | 0.74 | 0.76 | 0.75 | 0.31 |
| Echocardiographic documentation | 156 (4.5) | 0.85 | 0.78 | 0.79 | 0.79 | 0.08 |
| Holter monitor | 15 (0.4) | 0.80 | 0.73 | 0.78 | 0.78 | 0.01 |
